# Extended Spectrum Beta-Lactamase-Producing Enterobacteriaceae in International Travelers and Non-Travelers in New York City

**DOI:** 10.1371/journal.pone.0045141

**Published:** 2012-09-20

**Authors:** Scott A. Weisenberg, Jose R. Mediavilla, Liang Chen, Elizabeth L. Alexander, Kyu Y. Rhee, Barry N. Kreiswirth, Stephen G. Jenkins

**Affiliations:** 1 Department of Medicine, Alta Bates Summit Medical Center, Oakland, California, United States of America; 2 Public Health Research Institute, University of Medicine and Dentistry of New Jersey, Newark, New Jersey, United States of America; 3 Department of Medicine, Weill Cornell Medical College, New York, New York, United States of America; 4 Department of Pathology, Weill Cornell Medical College, New York, New York, United States of America; Mahidol Oxford Tropical Medicine Research Unit, Thailand

## Abstract

**Background:**

We performed this study 1) to determine the prevalence of community-associated extended spectrum beta-lactamase producing Enterobacteriaceae (ESBLPE) colonization and infection in New York City (NYC); 2) to determine the prevalence of newly-acquired ESBLPE during travel; 3) to look for similarilties in contemporaneous hospital-associated bloodstream ESBLPE and travel-associated ESBLPE.

**Methods:**

Subjects were recruited from a travel medicine practice and consented to submit pre- and post-travel stools, which were assessed for the presence of ESBLPE. Pre-travel stools and stools submitted for culture were used to estimate the prevalence of community-associated ESBLPE. The prevalence of ESBLPE-associated urinary tract infections was calculated from available retrospective data. Hospital-associated ESBLPE were acquired from saved bloodstream isolates. All ESBLPE underwent multilocus sequence typing (MLST) and ESBL characterization.

**Results:**

One of 60 (1.7%) pre- or non-travel associated stool was colonized with ESBLPE. Among community-associated urine specimens, 1.3% of *Escherichia coli* and 1.4% of *Klebsiella pneumoniae* were identified as ESBLPE. Seven of 28 travelers (25.0%) acquired a new ESBLPE during travel. No similarities were found between travel-associated ESBLPE and hospital-associated ESBLPE. A range of imported ESBL genes were found, including CTX-M-14 and CTX-15.

**Conclusion:**

ESBL colonization and infection were relatively low during the study period in NYC. A signficant minority of travelers acquired new ESBLPE during travel.

## Introduction

Extended spectrum beta-lactamase (ESBL) producing Enterobacteriaceae (ESBLPE) are an emerging cause of community-acquired infection worldwide [1]–[4], often resistant to standard antimicrobial choices. Intestinal colonization with ESBLPE provides a reservoir of bacteria that may cause infection of the host [5], be transmitted to others [6], and transmit resistance genes to other bacteria across species boundaries [7]. High rates of intestinal colonization with ESBLPE have been reported from many regions [8]–[10], although the prevalence in most communities in the United States is unknown. Risk factors for acquisition of ESBLPE are incompletely understood, but international travel has been associated with ESBLPE carriage [11], [12] and infection [13], [14]. We performed this study to: 1) characterize the prevalence of community-associated ESBLPE colonization and infection in New York City (NYC); 2) determine the frequency of acquisition of new ESBLPE during international travel; and 3) explore similarities between hospital-associated ESBLPE and any newly-imported ESBL-producing bacteria.

## Methods

### Subject Recruitment

Following Weill Cornell Medical College Institutional Review Board approval, subjects traveling to Central and South America, Africa, the Middle East, South Asia, Southeast Asia, and East Asia for 8 to 45 days were recruited from the Weill Cornell Travel Medicine (WCTM) practice in New York City between July 2009 and February 2010. Written informed consent was obtained from all subjects. Stool specimens were collected the week before travel and within a week upon return. Subjects were interviewed regarding travel itinerary, dietary habits during travel, health care exposure during or before travel, antibiotic use, and occurrence of traveler's diarrhea.

### Additional Study Samples and Data

A convenience sample of 20 stools submitted by non-travel associated outpatients was obtained from the Clinical Microbiology Laboratory at New York Presbyterian Hospital/Weill Cornell Medical College (NYPH/WCMC) to increase the sample size for NYC ESBLPE community prevalence estimation.

A separate convenience sample of ten archived ESBL-producing *E. coli* blood culture isolates cultured from inpatients from the same study period were obtained from the Clinical Microbiology Laboratory at NYPH/WCMC to look for similarities in MLST pattern and ESBL gene content between travel-associated and hospital-associated bacteria.

Records from the NYPH/WCMC Clinical Microbiology Laboratory were queried to determine the prevalence of ESBLPE amongst all community-associated urinary tract infections (UTI) during 2008. These were obtained from the laboratory database, and no additional testing of urinary ESBLPE was performed.

### Stool Analysis

Stools were stored at 4 degrees Celsius for up to 24 hours prior to processing. A swab from pre and post-travel stools, as well as the community stools, were selectively cultured for ESBLPE using MacConkey agar media containing cefpodoxime (4 µg/ml), followed by disk diffusion using ceftazidime and cefotaxime, with and without clavulanic acid, employing since-revised CLSI guidelines [15]. Positive (ATCC 700603) and negative (ATCC 35218) controls were used for quality control. VITEK-2 (bioMérieux Inc., Durham, NC) was used for species confirmation and antimicrobial susceptibility testing (using card AST-GN28). Pre-travel stool testing was repeated for all subjects with positive post-travel stools.

Multilocus sequence typing (MLST) was performed using the Pasteur *E. coli* MLST scheme maintained at http://www.pasteur.fr/recherche/genopole/PF8/mlst/ [16]. MLST sequence types (ST) of travel-acquired ESBLPE were compared with those of contemporary NYPH/WMC bloodstream infection isolates, as well as with each other. Any isolates meeting the since-revised CLSI criteria for ESBL production were subjected to PCR amplification of select ESBL genes using primers *bla*_TEM_ and *bla*_SHV_ [17], [18], and *bla*_CTX-M_ [19], [20]. Isolates were tested for the presence of carbapenemases using primers for *bla*_KPC_ [21], *bla*_IMP_, *bla*_VIM_, *bla*_OXA-48_ *bla*_NDM_ [22] and for *bla*_OXA-23,40,51,58_ [23]. Our primary interest was the presence or absence of *bla*_CTX-M_.

## Results

### Prevalence of ESBLPE in NYC Community Residents

Only one of 60 (1.7%) pre-travel or non-travel associated Enterobacteriaceae was classified as an ESBL producer. One of 40 (2.5%) subjects who returned a pre-travel stool was colonized by an ESBLPE, whereas none of 20 (0.0%) additional community-associated non-travel related stool samples were positive for ESBLPE.

### Prevalence of ESBLPE in Community UTIs

Twenty-seven of 2058 (1.3%) *E. coli* isolated from urine specimens submitted by outpatients were identified as ESBL producers by VITEK-2 in 2008. Seven of 486 (1.4%) *Klebsiella pneumoniae* isolates were identified as ESBL producers during the same period.

### Acquisition of ESBLPE during Travel

Twenty-eight subjects returned both pre-and post-travel stools. The median age was 66 (range 41–83), 19 (67.9%) were female, and all resided in Manhattan. Travel destinations included Central/South America (6), East/Southeast Asia (4), North Africa/Middle East (3), South Asia (7), and Sub-Saharan Africa (8). Travel duration ranged from 8–42 days (mean 16 days, median 14 days).

Seven of the 28 subjects (25.0%) harbored an ESBLPE (all *E. coli*) in the post-travel stool which was not present in the pre-travel stool. The only subject with an ESBL-producing *E. coli* in the pre-travel stool did not harbor ESBL-producing *E. coli* in the post-travel stool following travel to India. Acquisition of ESBL-producing *E. coli* occurred in travelers to South Asia (2/7), Central/South America (2/6), East/Southeast Asia (1/4), North Africa (1/3) and Sub-Saharan Africa (1/8). Among the 7 subjects who acquired an ESBL-producing *E. coli*, 1 experienced traveler's diarrhea, 1 took antibiotics during the trip, and none reported recent or “in trip” healthcare exposure. Dietary choices of the seven did not strictly follow the standard recommendation of “boil it, cook it, peel it, or forget it,” [24], but did not include raw or semi-raw meats. Lodging was comfortable to upscale, and one subject was visiting relatives in India.

### Analysis of ESBL-Producing E. coli Acquired During Travel

Among the 7 post-travel ESBLPE, 4 (57.1%) were susceptible *in vitro* to levofloxacin and gentamicin, 1 (14.3%) was susceptible to trimethoprim-sulfamethoxazole, and all were susceptible to cefepime, ertapenem, and meropenem (Table 1).

**Table 1:** Characteristics of Imported ESBLPE.

Travel Location	ST	ESBL genes	erta	t/s	levo	gent
Vietnam	39	CTX-M-14	S	S	S	S
Panama	8	CTX-M-14	S	R	S	S
Egypt	37	SHV-12	S	R	R	R
India	399	CTX-M-15	S	R	R	R
India	8	CTX-M-14	S	R	S	S
Uganda	437	CTX-M-15	S	R	S	S
Uruguay	83	not identified	S	R	R	R

The seven imported ESBLPE are shown. Legend: Travel = Destination Country; ST = multilocus sequence type (Pasteur *E. coli* MLST scheme); ESBL = extended spectrum beta-lactamase; erta = ertapenem; t/s = trimethoprim-sulfamethoxazole; levo = levofloxacin; gent = gentamicin; S = susceptible; R = resistant.

MLST of the post-travel ESBLPE showed that ESBL-producing *E. coli* acquired during travel to India and Panama belonged to the same sequence type (ST 8, Table 1). The remaining ESBLPE were unrelated to each other. None of the 7 ESBL-producing *E. coli* belonged to the pandemic ST43 clone (also known as ST131 in an alternative *E. coli* MLST scheme, http://mlst.ucc.ie/mlst/dbs/Ecoli).

ESBL genes were identified in 6 of the post-travel ESBLPE (Table 1). CTX-M-14 genes were identified in travelers to Vietnam, India, and Panama, while CTX-M-15 genes were found in travelers to Uganda and India. None of the strains tested contained tested *bla*_OXA_, *bla*_KPC_, *bla*_IMP_, *bla*_VIM_, or *bla*_NDM_ genes.

### Comparison of Travel-Associated ESBL-Producing E. coli with Contemporary NYPH/WCMC Bloodstream ESBL-Producing E.coli

No relatedness was found by MLST between post-travel ESBLPE and 10 ESBL-producing *E. coli* obtained from hospital bloodstream isolates. Hospital-associated ESBLPE did contain various ESBL genes, including CTX-M-15 (*n* = 5) and CTX-M-13 (*n* = 3), as well as TEM-120 (*n* = 1), and SHV-12 (*n* = 1). Two of the hospital-associated CTX-M-15 containing *E. coli* isolates belonged to the worldwide epidemic ST43 (ST131).

## Discussion

In recent years, ESBLPE, particularly those harboring CTX-M genes, have spread worldwide [25], although the mechanisms of transmission are incompletely understood. Knowledge of local prevalence, as well as of factors associated with increased risk for ESBLPE infection in individual patients, is critical for rational clinical decision making. In this study, the prevalence of ESBL colonization and infection in NYC was relatively low, suggesting that ESBLPE were not highly prevalent in the NYC community during the study period. At the same time, the discovery of CTX-M-15-harboring ST43 (ST131) *E. coli* in hospital bloodstream isolates serves as a reminder that globally prevalent ESBLPE are present in NYC, and presumably elsewhere in the United States [26].

The relatively low prevalence of ESBLPE in our study population contrasts with many global regions wherein the prevalence of colonization with ESBLPE is significantly higher [27]. Since travelers become colonized by local Enterobacteriaceae within the first two weeks of travel [28], [29], travel to regions of high ESBLPE prevalence would be expected to result in colonization with ESBLPE. In fact, recent studies confirm international travel as a risk factor for infection with ESBLPE [14].

We found that 28% of the international travelers in this study acquired and imported ESBL-producing *E. coli* during travel. Our results complement and confirm those of several recent studies. Twenty-four percent of Swedish travelers acquired a new ESBLPE during travel in one study [30], while ESBLPE were found in 36% of Swedish patients with extra-European travel-associated diarrhea [12], 23% of Canadian travelers with diarrhea [11], and 18% of British travelers with diarrhea [31].

The importation of ESBLPE has a number of clinical and public health consequences. Individuals colonized with certain strains of ESBLPE appear more likely to suffer ESBLPE infection [5], while most patients with ESBLPE-associated infection are colonized with ESBLPE [6]. Moreover, household contacts of ESBLPE-infected patients are subject to increased risk of colonization by ESBLPE [6]. Commensal and pathogenic ESBLPE may also serve as a reservoir for ESBL resistance genes that can undergo horizontal transmission to other Enterobacteriaceae [7]. Finally, intestinal colonization among returning travelers may serve as a “sentinel” of antimicrobial resistance patterns in visited regions. While epidemiologically important ESBL genes were already present in NYC during the study period (as evidenced by our hospital ESBLPE), ongoing importation of travel associated ESBLPE importations my lead to increased prevalence of ESBLPE over time.

The study has several limitations. The number of returned stools was fairly low, although the prevalence of ESBLPE in post-travel stools is similar to what has been reported from other regions. We investigated for the presence of a limited number of ESBL genes, and did not identify the ESBL gene in one isolate. We employed two restricted populations to estimate the prevalence of ESBLPE colonization in NYC, whereas a more comprehensive city-wide screening may have uncovered areas with higher prevalence.

In summary, we found the prevalence of ESBLPE colonization and infection to be low compared with those of other global regions. Nevertheless, the ongoing importation of ESBL and other resistance genes through international travel and other methods suggests that the prevalence of community-associated ESBLPE may increase in NYC. Clinicians and public health workers need to maintain vigilance against this emerging threat in the care of patients and populations.
